# Supplementary material for: Fine-mapping of the HNF1B multicancer locus identifies candidate variants that mediate endometrial cancer risk
Source: Hum Mol Genet. 2014 Nov 6;24(5):1478–92. doi: 10.1093/hmg/ddu552 (PMC4321445; doi:10.1093/hmg/ddu552)
Supplement: Supplementary Data [file supp_24_5_1478__index.html]

Fine-mapping of the HNF1B multicancer locus identifies candidate variants that mediate endometrial cancer risk — Fine-mapping of the HNF1B multicancer locus identifies candidate variants that mediate endometrial cancer risk — Supplementary Data 

# Fine-mapping of the *HNF1B* multicancer locus identifies candidate variants that mediate endometrial cancer risk

## Supplementary Data

Supplementary Data

**Files in this Data Supplement:**

- Supplementary Data - Doc file
- Supplementary Table 1 - xlsx file
- Supplementary Table 2 - xlsx file
- Supplementary Table 3 - xlsx file
- Supplementary Table 4 - xlsx file
- Supplementary Table 5 - docx file
