# Supplementary material for: Fine-mapping of the HNF1B multicancer locus identifies candidate variants that mediate endometrial cancer risk
Source: Hum Mol Genet. 2014 Nov 6;24(5):1478–92. doi: 10.1093/hmg/ddu552 (PMC4321445; doi:10.1093/hmg/ddu552)
Supplement: Supplementary Data [file supp_ddu552_ddu552supp.doc]

**Supplementary Information for:**

**Fine-mapping of the *HNF1B* multicancer locus identifies candidate variants that mediate endometrial cancer risk**

Jodie N Painter^1^, Tracy A O’Mara^1^, Jyotsna Batra^2^, Timothy Cheng^3^, Felicity A Lose^1^, Joe Dennis^4^, Kyriaki Michailidou^4^, Jonathan P Tyrer^5^, Shahana Ahmed^5^, Kaltin Ferguson^1^, Catherine S Healey^5^, Susanne Kaufmann^1^, Kristine M Hillman^1^, Carina Walpole^2^, Leire Moya^2^, Pamela Pollock^6^, Angela Jones^3^, Kimberley Howarth^3^, Lynn Martin^3^, Maggie Gorman^3^, Shirley Hodgson^7^, National Study of Endometrial Cancer Genetics Group (NSECG)^3,8^, CHIBCHA Consortium^3,8^, Ma. Magdalena Echeverry de Polanco^9^, Monica Sans^10^, Angel Carracedo^11,12^, Sergi Castellvi-Bel^13^, Augusto Rojas-Martinez^14^, Erika Santos^15^, Manuel R Teixeira^16^, Luis Carvajal-Carmona^3,9,17^, Xiao-Ou Shu^18^, Jirong Long^18^, Wei Zheng^18^, Yong-Bing Xiang^19^, The Australian National Endometrial Cancer Study Group (ANECS)^1,8^, Grant W Montgomery^1^, Penelope M Webb^1^, Rodney J Scott^20-22^, Mark McEvoy^23^, John Attia^20,23^, Elizabeth Holliday^20,24^, Nicholas G Martin^1^, Dale R Nyholt^1^, Anjali K Henders^1^, Peter A Fasching^25,26^, Alexander Hein^26^, Matthias W Beckmann^26^, Stefan P Renner^26^, Thilo Dörk^27^, Peter Hillemanns^28^, Matthias Dürst^29^, Ingo Runnebaum^29^, Diether Lambrechts^30,31^, Lieve Coenegrachts^32^, Stefanie Schrauwen^32^, Frederic Amant^33^, Boris Winterhoff^34^, Sean C Dowdy^34^, Ellen L Goode^35^, Attila Teoman^34^, Helga B Salvesen^36,37^, Jone Trovik^36,37^, Tormund S Njolstad^36,37^, Henrica MJ Werner^36,37^, Katie Ashton^21,38^, Tony Proietto^39^, Geoffrey Otton^39^, Gerasimos Tzortzatos^40^, Miriam Mints^40^, Emma Tham^41^, RENDOCAS^8,41^, Per Hall^42^, Kamila Czene^42^, Jianjun Liu^43^, Jingmei Li^43^, John L Hopper^44^, Melissa C Southey^45^, Australian Ovarian Cancer Study (AOCS)^1,8,46^, Arif B Ekici^47^, Matthias Ruebner^26^, Nicola Johnson^48^, Julian Peto^49^, Barbara Burwinkel^50,51^, Frederik Marme^50,52^, Hermann Brenner^53,54^, Aida K Dieffenbach^53,54^, Alfons Meindl^55^, Hiltrud Brauch^56^, The GENICA Network^8,51–56^, Annika Lindblom^41^, Jeroen Depreeuw^30^, Matthieu Moisse^30^, Jenny Chang-Claude^62^, Anja Rudolph^63^, Fergus J Couch^64^, Janet E Olson^35^, Graham G Giles^44,65,66^, Fiona Bruinsma^65^, Julie M Cunningham^64^, Brooke L. Fridley^67^, Anne-Lise Børresen-Dale^68,69^, Vessela N Kristensen^68–70^, Angela Cox^71^, Anthony J Swerdlow^72,73^, Nicholas Orr^73^, Manjeet K Bolla^4^, Qin Wang^4^, Rachel Palmieri Weber^74^, Zhihua Chen^75^, Mitul Shah^5^, Juliet D French^1^, Paul D P Pharoah^5^, Alison M Dunning^5^, Ian Tomlinson^3^, Douglas F Easton^4,5^, Stacey L Edwards^1^, Deborah J Thompson^5^, Amanda B Spurdle*^1^

1. QIMR Berghofer Medical Research Institute, Brisbane, QLD, Australia

2. Australian Prostate Cancer Research Centre-Qld, Institute of Health and Biomedical Innovation, and School of Biomedical Science, Queensland University of Technology, Brisbane, QLD, Australia

3. Wellcome Trust Centre for Human Genetics, University of Oxford, Oxford, UK

4. Centre for Cancer Genetic Epidemiology, Department of Public Health and Primary Care, University of Cambridge, Cambridge, UK

5. Centre for Cancer Genetic Epidemiology, Department of Oncology, University of Cambridge, Cambridge, UK

6. Institute of Health and Biomedical Innovation and School of Biomedical Science, Queensland University of Technology, Brisbane, QLD, Australia

7. Department of Clinical Genetics, St George’s Hospital Medical School, London, UK

8. See Supplementary Information for full list of members

9. Grupo de investigación Citogenética, Filogenia y Evolución de Poblaciones, Universidad del Tolima, Ibagué, Tolima, Colombia

10. Department of Biological Anthropology, College of Humanities and Educational Sciences, University of the Republic, Magallanes, Montevideo, Uruguay

11. Grupo de Medicina Xenómica, Fundación Galega de Medicina Xenómica (SERGAS) and CIBERER- Universidade de Santiago de Compostela- Santiago de Compostela, Spain

12. Center of Excellence in Genomic Medicine Research, King Abdulaziz University, Jeddah, KSA

13. Genetic Predisposition to Colorectal Cancer Group, Gastrointestinal & Pancreatic Oncology Team, IDIBAPS/CIBERehd/Hospital Clínic,Centre Esther Koplowitz (CEK), Barcelona, Spain

14. Universidad Autónoma de Nuevo León, Pedro de Alba s/n, San Nicolás de Los Garza, Nuevo León, Mexico

15. Hospital A.C. Camargo, São Paulo, Brazil

16. Department of Genetics, Portuguese Oncology Institute, Porto, Portugal, and Biomedical Sciences Institute (ICBAS), University of Porto, Porto, Portugal

17. Genome Center and Department of Biochemistry and Molecular Medicine, University of California, Davis, CA, USA

18. Department of Medicine, Vanderbilt Epidemiology Center, Vanderbilt-Ingram Cancer Center, Vanderbilt University Medical Center, Nashville, TN, USA

19. Department of Epidemiology, Shanghai Cancer Institute, Shanghai, China

20. Hunter Medical Research Institute, John Hunter Hospital, Newcastle, NSW, Australia

21. Hunter Area Pathology Service, John Hunter Hospital, Newcastle, NSW, Australia

22. Centre for Information Based Medicine and School of Biomedical Science and Pharmacy, University of Newcastle, NSW, Australia

23. Centre for Clinical Epidemiology and Biostatistics, School of Medicine and Public Health, University of Newcastle, NSW, Australia

24. Centre for Information Based Medicine and School of Medicine and Public Health, University of Newcastle, NSW, Australia

25. University of California at Los Angeles, Department of Medicine, Division of Hematology/Oncology, David Geffen School of Medicine, Los Angeles, CA, USA

26. University Hospital Erlangen, Friedrich-Alexander University Erlangen-Nuremberg, Erlangen, Germany

27. Hannover Medical School, Gynaecology Research Unit, Hannover, Germany

28. Hannover Medical School, Clinics of Gynaecology and Obstetrics, Hannover, Germany

29. Friedrich Schiller University Jena, Dept. of Gynaecology, Jena, Germany

30. Vesalius Research Center, VIB, Leuven, Belgium

31. Laboratory for Translational Genetics, Department of Oncology, KU Leuven, Belgium

32. Department of Oncology, KU Leuven, Belgium

33. Division of Gynaecological Oncology, Department of Oncology, University Hospital Leuven, KU Leuven, Belgium

34. Department of Obstetrics and Gynecology, Division of Gynecologic Oncology, Mayo Clinic, Rochester, MN, USA

35. Department of Health Science Research, Division of Epidemiology, Mayo Clinic, Rochester, MN, USA

36. Centre for Cancerbiomarkers, Department of Clinical Science, The University of Bergen, Norway

37. Department of Obstetrics and Gynecology, Haukeland University Hospital, Bergen, Norway

38. Centre for Information Based Medicine and the Discipline of Medical Genetics, School of Biomedical Sciences and Pharmacy, Faculty of Health, University of Newcastle, NSW, Australia

39. School of Medicine and Public Health, Faculty of Health, University of Newcastle, NSW, Australia

40. Department of Women’s and Children's Health, Karolinska Institutet, Karolinska University Hospital, Stockholm, Sweden

41. Department of Molecular Medicine and Surgery, Karolinska Institutet, Stockholm, Sweden

42. Department of Medical Epidemiology and Biostatistics, Karolinska Institutet, Stockholm, Sweden

43. Human Genetics, Genome Institute of Singapore, Singapore, Sweden

44. Centre for Epidemiology and Biostatistics, Melbourne School of Population and Global Health, The University of Melbourne, VIC, Australia

45. Genetic Epidemiology Laboratory, Department of Pathology, The University of Melbourne, Melbourne, VIC, Australia

46. Peter MacCullum Cancer Centre, Melbourne, VIC, Australia

47. Institute of Human Genetics, University Hospital, Erlangen, Friedrich-Alexander University Erlangen-Nuremberg, Erlangen, Germany.

48. Breakthrough Breast Cancer Research Centre, Institute of Cancer Research, London, UK

49. London School of Hygiene and Tropical Medicine, London, UK

50. Molecular Biology of Breast Cancer, Department of Gynecology and Obstetrics, University of Heidelberg, Heidelberg, Germany

51. Molecular Epidemiology, C080, German Cancer Research Center, DKFZ, Heidelberg, Germany

52. National Center for Tumor Diseases, University of Heidelberg, Heidelberg, Germany

53. Division of Clinical Epidemiology and Aging Research, German Cancer Research Center (DKFZ), Heidelberg, Germany

54. German Cancer Consortium (DKTK), Heidelberg, Germany

55. Department of Obstetrics and Gynecology, Division of Tumor Genetics, Technical University of Munich, Munich, Germany

56. Dr. Margarete Fischer-Bosch Institute of Clinical Pharmacology Stuttgart, University of Tuebingen, Germany

57. Institute for Occupational Medicine and Maritime Medicine, University Medical Center Hamburg-Eppendorf, Germany

58. Department of Internal Medicine, Evangelische Kliniken Bonn gGmbH, Johanniter Krankenhaus, Bonn, Germany

59. Institute of Pathology, Medical Faculty of the University of Bonn, Bonn, Germany

60. Institute for Prevention and Occupational Medicine of the German Social Accident Insurance (IPA), Bochum, Germany

61. Molecular Genetics of Breast Cancer, Deutsches Krebsforschungszentrum (DKFZ), Heidelberg, Germany

62. Division of Cancer Epidemiology, German Cancer Research Center, Heidelberg, Germany

63. Department of Cancer Epidemiology/Clinical Cancer Registry and Institute for Medical Biometrics and Epidemiology, University Clinic Hamburg-Eppendorf, Hamburg, Germany

64. Departments of Laboratory Medicine and Pathology, and Health Science Research, Mayo Clinic, Rochester, MN, USA

65. Cancer Epidemiology Centre, The Cancer Council Victoria, Melbourne, VIC, Australia

66. Department of Epidemiology and Preventive Medicine, Monash University, Melbourne, VIC Australia

67. Department of Biostatistics, University of Kansas Medical Center, Kansas City, KS, USA

68. Department of Genetics, Institute for Cancer Research, The Norwegian Radium Hospital, Oslo, Norway

69. The K.G. Jebsen Center for Breast Cancer Research, Institute for Clinical Medicine, Faculty of Medicine, University of Oslo, Oslo, Norway

70. Department of Clinical Molecular Oncology, Division of Medicine, Akershus University Hospital, Ahus, Norway

71. Sheffield Cancer Research Centre, Department of Oncology, University of Sheffield, UK

72. Division of Genetics and Epidemiology, Institute of Cancer Research, London, UK

73. Division of Breast Cancer Research, Institute of Cancer Research, London, UK

74. Department of Community and Family Medicine, Duke University School of Medicine, Durham, NC, USA

75. Department of Cancer Epidemiology, Division of Population Sciences, Moffitt Cancer Center, Tampa, FL, USA

**** Correspondence to:*** Amanda B. Spurdle, QIMR Berghofer Medical Research Institute, 300 Herston Rd, Herston, 4006, Queensland, Australia.

Ph: +61 (0)7 3362 0379

Fax: +61 (0)7 3362 0105

Email: [Amanda.Spurdle@qimrberghofer.edu.au](mailto:Amanda.Spurdle@qimrberghofer.edu.au)

**INDEX**

A. Detailed Description of the Case and Control Sample Sets [6](#__RefHeading___Toc386216543)

B. Genotype, expression and methylation in endometrial cancer tumor samples [10](#__RefHeading___Toc386216544)

Supplementary Figure 1. Signal intensity cluster plot for rs7501939 from the iCOGS array genotyping of 4,402 endometrial cancer cases and 28,758 country-matched controls. [10](#__RefHeading___Toc386216545)

Supplementary Figure 2. Regional linkage disequilibrium to our top SNP rs11263763 for Caucasian, Asian and African individuals in the 1000Genomes pilot data (http://www.broadinstitute.org/mpg/snap/ldplot.php). [11](#__RefHeading___Toc386216546)

Supplementary Figure 3. *HNF1B* isoform expression. [14](#__RefHeading___Toc386216547)

C. References [16](#__RefHeading___Toc386216550)

D. ECAC Study Collaborators (for case samples): [18](#__RefHeading___Toc386216551)

E. BCAC and OCAC Study Collaborators (for control samples): [19](#__RefHeading___Toc386216552)

F. Supplementary Acknowledgements [19](#__RefHeading___Toc386216553)

G. Additional Acknowledgments of Funding to BCAC/OCAC control groups [21](#__RefHeading___Toc386216554)

**SUPPLEMENTARY INFORMATION**

## A. Detailed Description of the Case and Control Sample Sets

A summary of the studies included in the iCOGS fine-mapping dataset and the genome-wide association study (GWAS) datasets is shown in Supplementary Table 1, with additional details provided below. All studies were predominantly of women of European ancestry, with the exception of SECGS which was based on women of Asian ancestry from China. All studies have the relevant IRB approval in each country in accordance with the principles embodied in the Declaration of Helsinki, and informed consent was obtained from all participants. A total of 6,608 cases and 37,925 controls were included in the Caucasian meta-analysis, and an additional 834 cases and 1936 controls were available from the SECGS Asian sample set.

***Fine-mapping (iCOGS) Case Sample Sets:***

The iCOGS fine-mapping data set included cases from 9 studies detailed below, as well as additional European ancestry cases from ANECS and SEARCH (non-overlapping with the GWAS datasets).

**BECS**

The Bavarian Endometrial Cancer Cases and Controls Study (BECS) is a single-center case-control study, conducted between 2002 and 2008, with the aim of investigating genetic and epidemiological risk factors for endometrial cancer. Cases were either incident cases referred to the University Hospital Erlangen by surrounding practioners (66% of the case sample set), or prevalent cases that were outpatients in follow-up care approached within 6.2 (±4.6 SD) years after treatment for primary endometrial cancer in the same hospital (34% of the case sample set). Epidemiological information was collected by a structured questionnaire completed during an interview and clinical data for the cases was obtained from clinical health records.

**CAHRES**

Details of the population selection process have been published previously for the Cancer Hormone Replacement Epidemiology Study (CAHRES) (1). Formerly known as the Singapore and Sweden Breast/Endometrial Cancer Study (SASBAC), this population based case-control study was conducted among Swedish women aged 50-74 years, who were residing in Sweden between January 1^st^ 1994 and December 31^st^ 1995. Endometrial cancer cases were identified through the nation-wide cancer registries in Sweden. All participants provided detailed questionnaire information. For endometrial cancer, histological specimens were reviewed and re-classified by the study pathologist. All participants reported Caucasian ethnicity.

**HJECS**

The Hannover-Jena Endometrial Cancer Study (HJECS), a hospital-based case-control study, included 250 German women, aged 31-89 years, who were recruited either at the Friedrich Schiller University of Jena or at Hannover Medical School after having been diagnosed with histologically confirmed primary incident endometrial carcinoma between 2004 and 2010. Epidemiological data were obtained from questionnaires, and information on tumor stage and histology was obtained from pathology and clinical reports. Over 98% were of German descent. Interviews were conducted at either the Friedrich Schiller University of Jena or at Hannover Medical School, and peripheral blood was collected for the extraction of DNA from white blood cells.

**LES**

The Leuven Endometrial Study (LES) is a hospital based case-control study. Eligible cases, identified by active surveillance of electronic patient files at the Leuven University Hospital, were white women aged 27-80 years diagnosed with endometrial cancer. Clinical data for endometrial cancer patients were recorded during interview at the time of diagnosis, and from pathology reports. All medical records were reviewed by trained abstractors and pathology reports compatible with primary, invasive, epithelial endometrial adenocarcinoma of all stages (I –IV) and all grades were consulted. Participation rates exceeded 95% for cases.

**MECS**

The Mayo Endometrial Cancer Study (MECS) is a hospital based prospective biobank collection. The majority of patients seen at Mayo Clinic Rochester with primary endometrial cancer diagnosed at age 18 and older are enrolled. The collection was started in 2006 and contains blood and fresh frozen tissue. DNA was isolated from white blood cells using Qiagen isolation kits. DNA concentration was measured with picogreen. Clinical data were abstracted from electronic medical records. Control data were obtained from Mayo Clinic OCAC controls.

**MoMaTEC**

Molecular Markers in Treatment of Endometrial Cancer (MoMaTEC) cases were recruited from an unselected patient population primarily treated for endometrial carcinoma at Haukeland University Hospital, Bergen during 2001-2009. This is the referral hospital for Hordaland county; the area is demographically well defined, with about 450,000 inhabitants, representing approximately 10% of the Norwegian population and with a similar incidence rate and prognosis as the total Norwegian population of endometrial cancers (2-4). Clinical Information for cases regarding age, FIGO stage, histologic subtype, grade and prognosis was extracted from medical records.DNA was extracted from peripheral blood samples.

**NECS**

The Newcastle Endometrial Cancer Study (NECS) includes histologically confirmed endometrial cancer cases consecutively recruited from 1992 up to 2005 at the Hunter Centre for Gynaecological Cancer, John Hunter Hospital, Newcastle, New South Wales, Australia(5). The final analysis included 194 endometrial cancer patients. Data on reproductive and environmental risk factors including ethnicity, was collected using self-reported questionnaires. Information regarding recurrence, stage, grade and histology of endometrial cancer was collected from medical records. Patients presenting at this hospital-based site were captured by ANECS recruitment from 2005 onwards.

**NSECG**

National Study of the Genetics of Endometrial Cancer (NSECG) cases were identified from collaborating clinicians throughout the UK from 2008 to present, taking care not to recruit from centres involved in SEARCH. Inclusion criteria were adenocarcinomas of the uterus presenting at 70 years of age or younger. Almost all cases were incident and sampled within 6 months of diagnosis. Peripheral blood was collected from each participant and DNA extracted using standard methods. Tumor histology was confirmed from routine hospital reports and further details of histopathology and other tumor pathology characteristic was abstracted from these clinical pathology reports. A sample of 797 cases that were non-overlapping with the NSECG GWAS were genotyped using the iCOGS chip, as indicated in Supplementary Table 1.

**RENDOCAS**

The Registry of Endometrial Cancer in Sweden (RENDOCAS) is a hospital based case-control study. Patients (n=262) who underwent surgery for endometrial cancer at Karolinska University hospital Solna, Sweden between 2008 and 2011 were included in the study. For each patient, the following was collected: blood and tumor samples; detailed family history and formulation of a pedigree where all suspected cancer cases were verified in medical records/pathology report if possible; questionnaire covering relevant environmental factors underlying endometrial cancer.

**Control sample sets**

As indicated in **Supplementary Table 1**, endometrial cancer case sample sets were matched by country to combined control sample sets from the same country that had been genotyped using the iCOGS chip. Data was largely from control sample sets that participated in the Breast Cancer Association Consortium iCOGS experiment (6), with iCOGS data also accessed for controls from the Mayo Clinic via the Ovarian Cancer Association Consortium (MAY) (7). In addition, iCOGS genotyping was performed for 183 Norwegian female controls, recruited in Bergen via the blood bank specifically for use in the MoMaTEC case-control genotyping studies.

***GWAS Case and Control Sample Sets:***

**ANECS**

The Australian National Endometrial Cancer Study (ANECS) is an Australian population-based case-control family study of cancer of the uterine corpus (8). Women aged 18-79, newly diagnosed with histologically confirmed primary cancer of the endometrium between July 2005 and December 2007 were identified through major hospitals nationally, and also from state-based cancer registries. Excluding women who could not be contacted (mostly due to death, illness or failure to contact), case participation rate was 63%. Participants completed a detailed questionnaire providing clinical and epidemiological information, including ethnicity of all four grandparents. Information on tumor pathology characteristics was abstracted in standardized format from clinical pathology reports for all patients.

**SEARCH**

The Studies of Epidemiology and Risk factors in Cancer Heredity (SEARCH) is an ongoing population-based study with cases ascertained through the Eastern Cancer Registration and Information Centre ([http://www.ecric.org.uk](http://www.ecric.org.uk/)). All women diagnosed with endometrial cancer between the ages of 18-69 years (average age of diagnosis 58 years) from August 2001 to September 2007 were eligible for inclusion. Approximately 54% of eligible patients have enrolled in the study. Women taking part in the study were asked to provide a 20ml blood sample for DNA analysis, and to complete a comprehensive epidemiological questionnaire. Controls were also drawn from SEARCH (http://ccge.medschl.cam.ac.uk/search/), but had no prior history of cancer at the time of recruitment. They were female, also between the ages of 18-69 at the time of recruitment and matched to cases in geographical profile. Approximately 35% of eligible controls enrolled in the study. All participants reported Caucasian ethnicity. Information on tumor pathology characteristics was provided by the Eastern Cancer Registration and Information Centre and was derived from clinical pathology reports for all patients.

Genome-wide genotyping of the ANECS and SEARCH cases was performed using an Illumina Infinium 610K array and called using the Illuminus algorithm. Genotypes were available for 1317 cases with endometrial cancer. Samples were excluded as follows: probable Turner’s syndrome or male sex based on genotypes for markers on the X and Y chromosomes (n=4); call rate <95% (n=15); heterozygosity outside 5 standard deviations from the mean (n=7); probable sibling pairs identified as close relatives by identity-by-state probabilities >0.85 (n=3); >15% non-European ancestry estimated from identity-by-state scores (n=1), leaving a total of 1287 cases (606 from ANECS and 681 from SEARCH). The duplicate concordance was 99.998%.

**QIMR**

The QIMR Berghofer Medical Research Institute control sample (9) is a subsection of individuals recruited as part of the Brisbane Adolescent Twin Study (10, 11). Twins were recruited from schools in Brisbane, Australia and surrounding areas of southeast Queensland and were examined close to their 12th birthday. Blood was obtained from all twins and most parents. Parents were asked the ancestry of all eight great-grandparents of the twins. More than 95% of great-grandparents were identified as being of northern European ancestry, mainly from Britain and Ireland. This analysis used genotype data from parents and siblings only, extracted from an existing Illumina 610K BeadChip genome-wide association scan (9) and recalled using the Illuminus algorithm. After standard QC steps (as for the case data) 1,846 QIMR Berghofer controls were included in the analysis.

**HCS**

The Hunter Community Study (HCS) is a population-based cohort study consisting of men and women aged 55-85 years of age in Newcastle, New South Wales, Australia (12). Participants were randomly selected from the NSW State electoral roll (listing on the electoral roll is compulsory in Australia) and contacted between December 2004 and December 2007. Non-English speaking persons and those living in a residential aged-care facility were ineligible for participation in the study. Participants were asked to complete five self-report questionnaires as well as attend the HCS data collection centre so clinical measures could be obtained. In total, 44.5% of eligible controls agreed to participate in this study. Genotype data for this study were extracted from an existing Illumina 610K BeadChip genome-wide association study scan and recalled using the Illuminus algorithm. After standard QC steps (as for the case data) 1,237 HCS controls were included in the analysis.

**WTCCC**

Controls utilized for stage 1 analysis were genotyped as part of the Wellcome Trust Case Control Consortium (WTCCC2) (13). These controls are drawn from two sources: 2,922 controls from the 1958 Birth Cohort (1958BC), a population-based study in the United Kingdom of individuals born in 1 week in 1958 (14); and 2,737 controls identified through the UK National Blood Service (NBS) (13). The analyses presented here are based on 2,694 1958BC and 2,496 NBS controls for which valid genotype data were available at the time of analysis.

**NSECG**

As detailed above, NSECG cases were identified from collaborating clinicians throughout the UK, based on diagnosis of adenocarcinoma of the uterus at 70 years of age or younger. A sample of 919 non-overlapping NSECG cases were genotyped at the Wellcome Trust Centre for Human Genetics Oxford using the Illumina 660K genome-wide array. Controls were spouses or partners of colorectal cancer cases unaffected by cancer and without a personal family history (to 2^nd^ degree relative level) of colorectal neoplasia drawn from the UK1/CORGI colorectal cancer sample set, genotyped previously using the Illumina 550K genome-wide array (15).

**SECGS Asian dataset**

The Shanghai Endometrial Cancer Genetic Study (SECGS) includes 834 endometrial cancer cases who were recruited to the Shanghai Endometrial Cancer Study (SECS) and 1936 controls who were recruited to the Shanghai Breast Cancer Study (SBCS). As described in detail elsewhere, both SECS and SBCS are two population-based case-control studies that were conducted in parallel in Shanghai during same period using an identical study protocol (16, 17). Briefly, 1,199 women aged between 30 and 69 with newly diagnosed with EC between 1997 and 2003 were identified through the population-based tumor registry and recruited to the SECS (response rate 83%). The SBCS controls were randomly selected from the general population using the Shanghai Resident Registry with response rate of 74%. Women with prior hysterectomies were not eligible for inclusion in this study. Participants completed a detailed in-person interview at the time of enrollment and provided a blood or buccal cell sample. Case and control genotype data for stage 2 SNPs, or for correlated SNPs with *r^2^*>0.8, were extracted from existing Affymetrix 6.0 genome-wide scan data (18).

## B. Genotype, expression and methylation in endometrial cancer tumor samples

## Supplementary Figure 1. Signal intensity cluster plot for rs7501939 from the iCOGS array genotyping of 4,402 endometrial cancer cases and 28,758 country-matched controls.


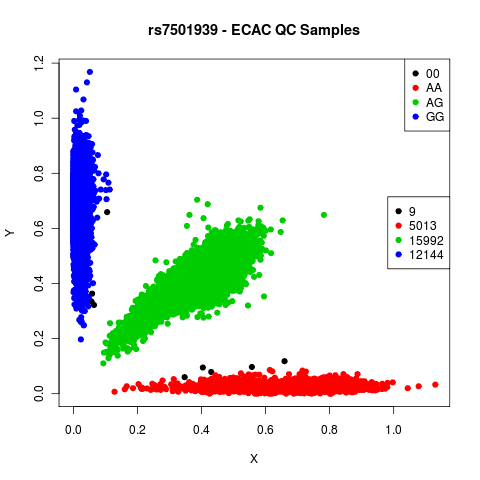


## Supplementary Figure 2. Regional linkage disequilibrium to our top SNP rs11263763 for Caucasian, Asian and African individuals in the 1000Genomes pilot data ([**http://www.broadinstitute.org/mpg/snap/ldplot.php**](http://www.broadinstitute.org/mpg/snap/ldplot.php)).

| **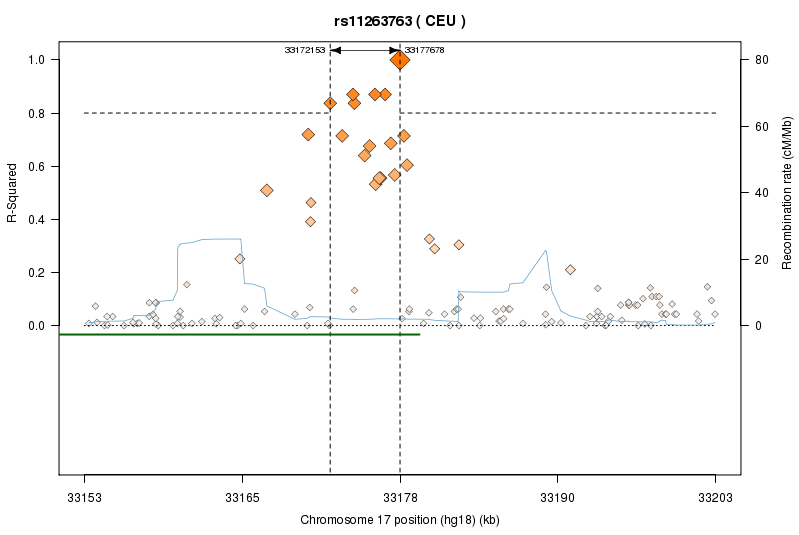** |
| --- |
| **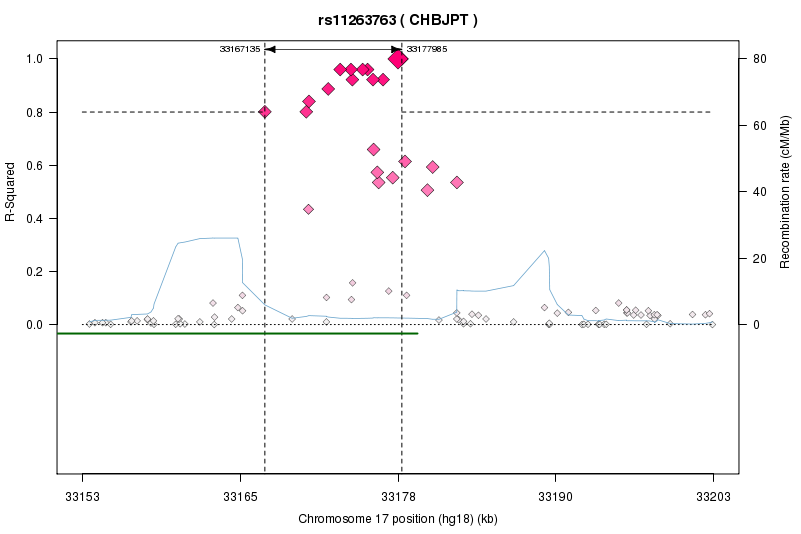** |
| **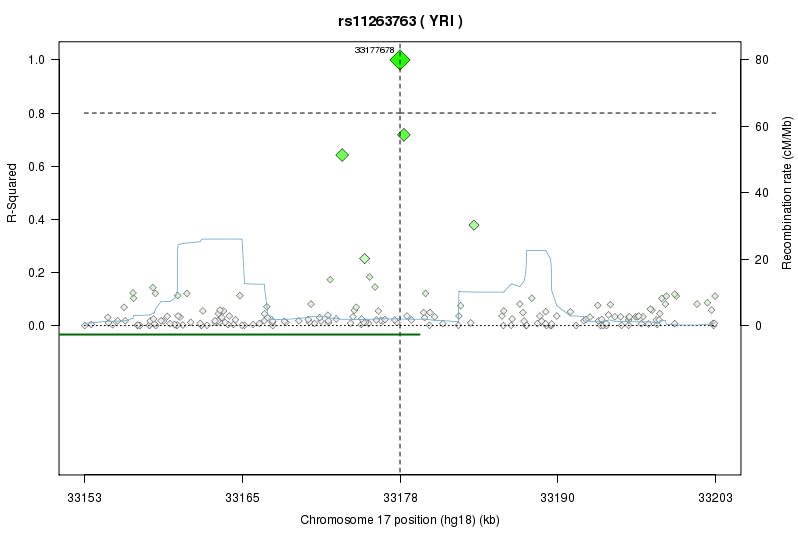** |

**Expression and methylation analyses**

SNP rs4430796 was recently reported as an an eQTL in benign prostate tissue (19), with the presence of the minor ‘G’ allele significantly decreasing *HNF1B* expression in histologically normal prostate tissue from European American, African American and Japanese patients. The eQTL effect was consistent although not significant in tumor tissue, however the sample sizes were typically smaller (19). Conversely, expression levels in ovarian cancer appear to be mediated through epigenetic mechanisms (7, 20). In invasive serous tumors *HNF1B* expression is typically absent, with high levels of promoter methylation in ~42% of tumors (with another epigenetic mechanism proposed to influence *HNF1B* expression in the remaining tumors), while *HNF1B* is expressed in most clear cell ovarian tumors concurrent with typically unmethylated *HNF1B* promoters (20). SNP rs757210, associated with both serous and clear cell ovarian cancer risk, is not associated with *HNF1B* expression but is associated with *HNF1B* methylation in serous ovarian cancer (7).

To begin investigating whether the decreased risk of endometrial cancer mediated through rs1163763 (and/or SNPs in LD with it) resembles associations seen in either prostate or clear cell ovarian cancer (as *HNF1B* methylation is typically absent in endometrial cancer tumors) we mined various datasets generated by us for our ANECS samples, and also The Cancer Genome Atlas (TCGA: <https://tcga-data.nci.nih.gov/tcga/>).

***The Cancer Genome Atlas data***

Level 3 (processed) data from endometrial tumors is publically available through TCGA (21). Analyses were restricted to incorporate data only from tumor samples of endometrioid endometrial cancer (type I, the most common form) with no evidence of copy-number alterations taken from individuals of Caucasian ancestry. SNP genotypes (Affymetrix 6.0 arrays) were downloaded through the controlled access portal, while epidemiological data, normalized RNA_Seq data (Illumina GAIIx) and DNA methylation data (Illumina Infinium HumanMethylation 450 Beadchips) were downloaded through the public access TCGA portal. RNA-Seq ZScores, tumor categories and GISTIC copy number calls for TCGA samples were obtained from the cBio Portal for Cancer Genomics (<http://www.cbioportal.org/public-portal/index.do>) and compared back to the TCGA data to confirm relative gene expression, tumor category and copy number assignments.

***SNP data***

Level 2 (preprocessed) germline GWAS data from endometrial cancer patients was downloaded from the TCGA data portal and the following QC performed. SNPs were excluded for call rate <95%, MAF <99% or deviations from HWE significant at 10^-4^. Samples were excluded for low overall call rate (<95%), heterozygosity >3 standard deviations from the mean, inconclusive sex status (X-chromosome homozygosity rate between 0.2 and 0.8), or samples >6 standard deviations from the mean scores for principal component 1 or 2, calculated using CEU individuals in HapMap (<http://hapmap.ncbi.nlm.nih.gov/>). For duplicate samples or samples identified as close relatives by IBS probabilities >0.85, the sample with the lower call rate was excluded.

***Gene expression (eQTL analysis)***

*HNF1B mRNA expression by genotype, and molecular tumor type*

Genotypes for SNPs located between chr17: 35,599,377-36,602,919 on the Affymetrix 6.0 array and normalized RNA_Seq gene expression data were available for 213 tumor samples. There were significant associations observed between the rs11263763 and rs11658063 genotypes and gene expression for the *HNF1B* gene (Kruskal-Wallis *P*=1.3x10^-2^ and 5.0x10^-3^ respectively), but not for any other gene located within 1Mb of *HNF1B* (data not shown). There was also no association between *HNF1B* expression and tumor molecular phenotype category, as defined by TCGA (*P*=0.47), namely: copy number high, copy number low, microsatellite instability hypermutated, and POLEultramutated.

*Isoform-specific expression*

Genetic databases show a number of different *HNF1B* transcripts and isoforms. A previous study in prostate cancer showed a significant shift in isoform usage between benign prostate and tumor tissue, with isoform C predominating in benign tissue and isoform B predominating in malignant tissue (22). We therefore tested this suggestion that isoform expression differences could influence cancer risk at this locus in the endometrial cancer cell lines. Contrary to prostate cancer, no significant down-regulation of isoform C was observed in tumor cell lines when compared to a normal cell line (Supplementary Fig 3).

Further, we investigated the association between genotype and *HNF1B* isoform expression in the TCGA data. This dataset provides normalized RNA_Seq data for 3 isoforms A (uc010wdi.1), B (uc002hok.3), and C (uc010cve.1). It should be noted that uc010cve.1 is not equivalent to the isoform C described above (uc010cve.1 includes only exons 3 and an extended exon 4), although expression of this transcript is likely to also capture the longer isoform C tested above. There was no significant association between genotype and isoform usage (*P*=0.45); rs11263763 genotype was significantly associated with expression of isoform B (uc002hok.3; *P*=2.2x10^-2^) and isoform A (uc010wdi.1; *P*=2.1x10^-2^), while isoform C (uc010cve.1) was expressed at an extremely low level in those samples in which expression was detected.

## Supplementary Figure 3. *HNF1B* isoform expression. A. RT-PCR results using *HNF1B* isoform-specific primers. i. *HNF1B* isoforms A and B; ii. *HNF1B* isoform C as described by Harries *et al*. (including exons 1-4) (22); iii. *HPRT1* positive control. Lanes 1-10 are endometrial cell lines (lane 2 is the immortalized normal cell line); NTC non-template control. LNCaP prostate cancer cell line. 2% agarose gels. DNA Molecular Weight Marker (0.07-12.2Kbp). B. *HNF1B* isoforms included in the TCGA dataset, where isoform A corresponds to uc010wdi.1, B corresponds to uc002hok.3, and C corresponds to uc010cve.1 (distinct from the Harries *et al.* (22) isoform C described above).

**A.**


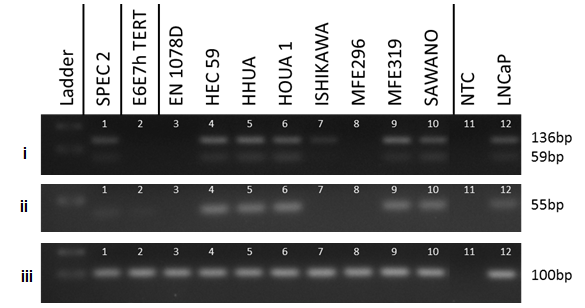


**B.**

***
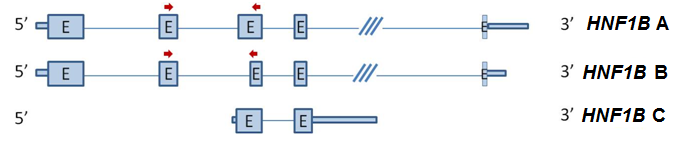
***

***Gene expression by DNA methylation analysis.***

Gene expression (RNA-Seq) and *HNF1B* methylation data were available for 199 tumor samples. Investigations into *HNF1B* methylation in ovarian tumors have focused on Illumina probe cg14487292 (7, 20), however, data for this probe was missing from the TCGA endometrial cancer data. Instead, we analysed average methylation across 18 probes located with the region of the *HNF1B* CpG island (cg15246719, cg12134754, cg24712484, cg03433642, cg09679923, cg17652435, cg03348978, cg02435495, cg05110178, cg11862993, cg09463047, cg04917276, cg04433035, cg05222347, cg02335804, cg12788467, cg13230606, and cg19378036), all of which had beta values suggesting very low /no methylation (beta values <0.2) for the majority of tumor samples (average beta across all 18 probes = 0.098). There was a significant negative correlation between methylation at these probes and HNF1B expression (Pearson’s r= -0.358, *P*=2.1x10^-7^). As above, there was also no association between *HNF1B* methylation and tumor category as defined by TCGA tumor subtype (*P*=0.78). Additionally, there was no association between rs11263763 or rs116658063 genotype with *HNF1B* methylation (*P*=0.42 and 0.37 respectively) or *MLH1* methylation at ‘region C’ probes (23) cg11600697, cg21490561 and cg00893636 (*P*=0.58 and 0.22 respectively).

**rs4430796 genotype and *MLH1* methylation in ANECS samples**

Additionally, data was available for 182 of our ANECS samples for both *MLH1* methylation, performed as part of a larger project to investigate the methylation profile of this gene in relation to endometrial tumor MMR protein expression and germline MMR gene mutation status(24), and genotype at the original GWAS SNP rs4430796, performed as part of the initial follow-up of the association at the *HNF1B* locus (25). Of these samples, 86 showed *MLH1* methylation while 96 showed no methylation at this locus. There was no association between rs4430796 genotype and methylation status in these samples (*P*=0.91).

## C. References

1 Weiderpass, E., Adami, H.O., Baron, J.A., Magnusson, C., Bergstrom, R., Lindgren, A., Correia, N. and Persson, I. (1999) Risk of endometrial cancer following estrogen replacement with and without progestins. *J Natl Cancer Inst*, **91**, 1131-1137.

2 Wik, E., Trovik, J., Iversen, O.E., Engelsen, I.B., Stefansson, I.M., Vestrheim, L.C., Haugland, H.K., Akslen, L.A. and Salvesen, H.B. (2009) Deoxyribonucleic acid ploidy in endometrial carcinoma: a reproducible and valid prognostic marker in a routine diagnostic setting. *Am J Obstet Gynecol*, **201**, 603 e601-607.

3 Salvesen, H.B., Iversen, O.E. and Akslen, L.A. (1999) Prognostic significance of angiogenesis and Ki-67, p53, and p21 expression: a population-based endometrial carcinoma study. *J Clin Oncol*, **17**, 1382-1390.

4 Salvesen, H.B., Carter, S.L., Mannelqvist, M., Dutt, A., Getz, G., Stefansson, I.M., Raeder, M.B., Sos, M.L., Engelsen, I.B., Trovik, J. *et al.* (2009) Integrated genomic profiling of endometrial carcinoma associates aggressive tumors with indicators of PI3 kinase activation. *Proc Natl Acad Sci U S A*, **106**, 4834-4839.

5 Ashton, K.A., Proietto, A., Otton, G., Symonds, I., McEvoy, M., Attia, J., Gilbert, M., Hamann, U. and Scott, R.J. (2008) The influence of the Cyclin D1 870 G>A polymorphism as an endometrial cancer risk factor. *BMC Cancer*, **8**, 272.

6 Michailidou, K., Hall, P., Gonzalez-Neira, A., Ghoussaini, M., Dennis, J., Milne, R.L., Schmidt, M.K., Chang-Claude, J., Bojesen, S.E., Bolla, M.K. *et al.* (2013) Large-scale genotyping identifies 41 new loci associated with breast cancer risk. *Nat Genet*, **45**, 353-361, 361e351-352.

7 Pharoah, P.D., Tsai, Y.Y., Ramus, S.J., Phelan, C.M., Goode, E.L., Lawrenson, K., Buckley, M., Fridley, B.L., Tyrer, J.P., Shen, H. *et al.* (2013) GWAS meta-analysis and replication identifies three new susceptibility loci for ovarian cancer. *Nat Genet*, **45**, 362-370, 370e361-362.

8 Spurdle, A. and Webb, P. (2008) Re: Excess of early onset multiple myeloma in endometrial cancer probands and their relatives suggests common susceptibility. *Gynecol Oncol*, **109**, 153; author reply 154.

9 Painter, J.N., Anderson, C.A., Nyholt, D.R., Macgregor, S., Lin, J., Lee, S.H., Lambert, A., Zhao, Z.Z., Roseman, F., Guo, Q. *et al.* (2011) Genome-wide association study identifies a locus at 7p15.2 associated with endometriosis. *Nat Genet*, **43**, 51-54.

10 McGregor, B., Pfitzner, J., Zhu, G., Grace, M., Eldridge, A., Pearson, J., Mayne, C., Aitken, J.F., Green, A.C. and Martin, N.G. (1999) Genetic and environmental contributions to size, color, shape, and other characteristics of melanocytic naevi in a sample of adolescent twins. *Genet Epidemiol*, **16**, 40-53.

11 Zhu, G., Duffy, D.L., Eldridge, A., Grace, M., Mayne, C., O'Gorman, L., Aitken, J.F., Neale, M.C., Hayward, N.K., Green, A.C. *et al.* (1999) A major quantitative-trait locus for mole density is linked to the familial melanoma gene CDKN2A: a maximum-likelihood combined linkage and association analysis in twins and their sibs. *Am J Hum Genet,* **65**, 483-492.

12 McEvoy, M., Smith, W., D'Este, C., Duke, J., Peel, R., Schofield, P., Scott, R., Byles, J., Henry, D., Ewald, B. *et al.* (2010) Cohort profile: The Hunter Community Study. *Int J Epidemiol*, **39**, 1452-1463.

13 Wellcome Trust Case Control Consortium. (2007) Genome-wide association study of 14,000 cases of seven common diseases and 3,000 shared controls. *Nature*, **447**, 661-678.

14 Power, C. and Elliott, J. (2006) Cohort profile: 1958 British birth cohort (National Child Development Study). *Int J Epidemiol*, **35**, 34-41.

15 Houlston, R.S., Cheadle, J., Dobbins, S.E., Tenesa, A., Jones, A.M., Howarth, K., Spain, S.L., Broderick, P., Domingo, E., Farrington, S. *et al.* (2010) Meta-analysis of three genome-wide association studies identifies susceptibility loci for colorectal cancer at 1q41, 3q26.2, 12q13.13 and 20q13.33. *Nat Genet*, **42**, 973-977.

16 Xu, W.H., Long, J.R., Zheng, W., Ruan, Z.X., Cai, Q., Cheng, J.R., Zhao, G.M., Xiang, Y.B. and Shu, X.O. (2009) Association of thymidylate synthase gene with endometrial cancer risk in a Chinese population. *Cancer Epidemiol Biomarkers Prev*, **18**, 579-584.

17 Zheng, W., Long, J., Gao, Y.T., Li, C., Zheng, Y., Xiang, Y.B., Wen, W., Levy, S., Deming, S.L., Haines, J.L. *et al.* (2009) Genome-wide association study identifies a new breast cancer susceptibility locus at 6q25.1. *Nat Genet*, **41**, 324-328.

18 Long, J., Zheng, W., Xiang, Y.B., Lose, F., Thompson, D., Tomlinson, I., Yu, H., Wentzensen, N., Lambrechts, D., Dork, T. *et al.* (2012) Genome-wide association study identifies a possible susceptibility locus for endometrial cancer. *Cancer Epidemiol Biomarkers Prev*, **21**, 980-987.

19 Grisanzio, C., Werner, L., Takeda, D., Awoyemi, B.C., Pomerantz, M.M., Yamada, H., Sooriakumaran, P., Robinson, B.D., Leung, R., Schinzel, A.C. *et al.* (2012) Genetic and functional analyses implicate the NUDT11, HNF1B, and SLC22A3 genes in prostate cancer pathogenesis. *Proc Natl Acad Sci U S A*, **109**, 11252-11257.

20 Shen, H., Fridley, B.L., Song, H., Lawrenson, K., Cunningham, J.M., Ramus, S.J., Cicek, M.S., Tyrer, J., Stram, D., Larson, M.C. *et al.* (2013) Epigenetic analysis leads to identification of HNF1B as a subtype-specific susceptibility gene for ovarian cancer. *Nat Commun*, **4**, 1628.

21 The Cancer Genome Atlas Research Network. (2013) Integrated genomic characterization of endometrial carcinoma. *Nature*, **497**, 67-73.

22 Harries, L.W., Perry, J.R., McCullagh, P. and Crundwell, M. (2010) Alterations in LMTK2, MSMB and HNF1B gene expression are associated with the development of prostate cancer. *BMC Cancer*, **10**, 315.

23 Metcalf, A.M. and Spurdle, A.B. (2013) Endometrial tumour BRAF mutations and MLH1 promoter methylation as predictors of germline mismatch repair gene mutation status: a literature review. *Familial cancer*, in press.

24 Buchanan DD, T.Y., Walsh MD, Clendenning M, Metcalf AM, Ferguson K, Arnold ST, Thompson BA, Lose FA, Parsons MT, Walters RJ, Pearson S-A, Cummings M, Oehler MK, Blomfield PB, Quinn MA, Kirk JA, Stewart CJ, Obermair A, Young JP, Webb PM, Spurdle AB. Tumor mismatch repair immunohistochemistry and DNA MLH1 methylation testing of patients with endometrial cancer diagnosed at age < 60 Years optimizes triage for population-level germline mismatch repair gene mutation testing. *J Clin Oncol*, **32**, 90-100.

25 Spurdle, A.B., Thompson, D.J., Ahmed, S., Ferguson, K., Healey, C.S., O'Mara, T., Walker, L.C., Montgomery, S.B., Dermitzakis, E.T., Fahey, P. *et al.* (2011) Genome-wide association study identifies a common variant associated with risk of endometrial cancer. *Nat Genet*, **43**, 451-454.

## D. ECAC Study Collaborators (for case samples):

***The ANECS Group comprises****:* AB Spurdle, PM Webb, J Young (QIMR Berghofer Medical Research Institute); Consumer representative: L McQuire; Clinical Collaborators: NSW: S Baron-Hay, D Bell, A Bonaventura, A Brand, S Braye, J Carter, F Chan, C Dalrymple, A Ferrier (deceased), G Gard, N Hacker, R Hogg, R Houghton, D Marsden, K McIlroy, G Otton, S Pather, A Proietto, G Robertson, J Scurry, R Sharma, G Wain, F Wong; Qld: J Armes, A Crandon, M Cummings, R Land, J Nicklin, L Perrin, A Obermair, B Ward; SA: M Davy, T Dodd, J Miller, M Oehler, S Paramasivum, J Pierides, F Whitehead; Tas: P Blomfield, D Challis; Vic: D Neesham, J Pyman, M Quinn, R Rome, M Weitzer; WA: B Brennan, I Hammond, Y Leung, A McCartney (deceased), C Stewart, J Thompson; Project Managers: S O'Brien, S Moore; Laboratory Manager: K Ferguson; Pathology Support: M Walsh; Admin Support: R Cicero, L Green, J Griffith, L Jackman, B Ranieri; Laboratory Assistants: M O'Brien, P Schultz; Research Nurses: B Alexander, C Baxter, H Croy, A Fitzgerald, E Herron, C Hill, M Jones, J Maidens, A Marshall, K Martin, J Mayhew, E Minehan, D Roffe, H Shirley, H Steane, A Stenlake, A Ward, S Webb, J White.

***CHIBCHA (study of hereditary cancer in Europe and Latin America) collaborators include:*** Ma. Magdalena Echeverry de Polanco, Mabel Elena Bohórquez, Rodrigo Prieto, Angel Criollo, Carolina Ramírez, Ana Patricia Estrada, Jhon Jairo Suárez (Grupo de Citogenética Filogenia y Evolución de Poblaciones, Universidad del Tolima, Colombia); Augusto Rojas Martinez (Center for Research and Development in Health Sciences, Universidad Autónoma de Nuevo León, Monterrey, Mexico); Silvia Rogatto, Samuel Aguiar Jnr, Ericka Maria Monteiro Santos (Department of Urology, School of Medicine, UNESP - São Paulo State University, Botucatu, Brazil); Monica Sans, Valentina Colistro, Pedro C. Hidalgo, Patricia Mut (Department of Biological Anthropology, College of Humanities and Educational Sciences, University of the Republic, Magallanes, Montevideo, Uruguay); Angel Carracedo, Clara Ruiz Ponte, Ines Quntela Garcia (Fundacion Publica Galega de Medicina Xenomica, CIBERER, Genomic Medicine Group-University of Santiago de Compostela, Hospital Clinico, Santiago de Compostela, Galicia, Spain); Sergi Castellvi-Bel (Department of Gastroenterology, Institut de Malalties Digestives i Metabòliques, Hospital Clínic, Centro de Investigación Biomédica en Red de Enfermedades Hepáticas y Digestivas, IDIBAPS, University of Barcelona, Barcelona, Catalonia, Spain); Manuel Teixeira (Department of Genetics, Portuguese Oncology Institute, Rua Dr, António Bernardino de Almeida, Porto, Portugal).

***NECS collaborators include:*** Ute Hamann and Michael Gilbert.

***The NSECG Group comprises:*** Ian Tomlinson (Oxford University); M Adams, A Al-Samarraie, S Anwar, R Athavale, S Awad, A Bali, A Barnes, G Cawdell, S Chan, K Chin, P Cornes, M Crawford, J Cullimore, S Ghaem-Maghami, R Gornall, J Green, M Hall, M Harvey, J Hawe, A Head, J Herod, M Hingorani, M Hocking, C Holland, T Hollingsworth,J Hollingworth, T Ind, R Irvine, C Irwin, M Katesmark, S Kehoe, G Kheng-Chew, K Lankester, A Linder, D Luesley, C B-Lynch,V McFarlane, R Naik, N Nicholas, D Nugent, S Oates, A Oladipo, A Papadopoulos, S Pearson, D Radstone, S Raju, A Rathmell, C Redman, M Rymer, P Sarhanis, G Sparrow, N Stuart, S Sundar, A Thompson, S Tinkler, S Trent, A Tristram, N Walji, R Woolas.

***RENDOCAS investigators include***: Annika Lindblom, Gerasimos Tzortzatos, Miriam Mints, Emma Tham, Ofra Castro, Kristina Gemzell-Danielsson.

***SEARCH collaborators include:*** Helen Baker, Caroline Baynes, Don Conroy, Bridget Curzon, Patricia Harrington, Sue Irvine, Craig Luccarini, Rebecca Mayes, Hannah Munday, Barbara Perkins, Daisy Pharoah, Radka Platte, Anne Stafford and Judy West.

***SECGS investigators include:***  Xiao Ou Shu, Wei Zheng, Jirong Long, Qiuyin Cai, Qi Dai, Hui Cai, Ryan Delahanty and Chun Li (Vanderbilt University), Yong-Bing Xiang, Yutang Gao, Wang-Hong Xu ( Shanghai Cancer Institute), Wei Lu, Ying Zheng, and Kai Gu (Shanghai Institute of Preventive Medicine).

## E. BCAC and OCAC Study Collaborators (for control samples):

***The Australian Ovarian Cancer Study Group comprises:*** R Stuart-Harris; NSW‐ F Kirsten, J Rutovitz, P Clingan, A Glasgow, A Proietto, S Braye, G Otton, J Shannon, T Bonaventura, J Stewart, S Begbie, M Friedlander, D Bell, S Baron-­Hay, A Ferrier (deceased), G Gard, D Nevell, N Pavlakis, S Valmadre, B Young, C Camaris, R Crouch, L Edwards, N Hacker, D Marsden, G Robertson, P Beale, J Beith, J Carter, C Dalrymple, R Houghton, P Russell, L Anderson, M Links, J Grygiel, J Hill, A Brand, K Byth, R Jaworski, P Harnett, R Sharma, G Wain; QLD- D Purdie, D Whiteman, B Ward, D Papadimos, A Crandon, M Cummings, K Horwood. A Obermair, L Perrin, D Wyld, J Nicklin; SA- M Davy, MK Oehler, C Hall, T Dodd, T Healy, K Pittman, D Henderson, J Miller, J Pierdes, A Achan; TAS- P Blomfield, D Challis, R McIntosh, A Parker; VIC- B Brown, R Rome, D Allen, P Grant, S Hyde, R Laurie M Robbie, D Healy, T Jobling, T Manolitsas, J McNealage, P Rogers, B Susil, E Sumithran, I Simpson, I Haviv, K Phillips, D Rischin, S Fox, D Johnson, S Lade, P Waring, M Loughrey, N O’Callaghan, B Murray, L Mileshkin, P Allan; V Billson, J Pyman, D Neesham, M Quinn, A Hamilton, C Underhill, R Bell, LF Ng, R Blum, V Ganju; WA- I Hammond, A McCartney (deceased), C Stewart, Y Leung, M Buck, N Zeps (WARTN); AOCS Management Group- DDL Bowtell, AC Green, G Chenevix-Trench, A deFazio, D Gertig, PM Webb.

***BSUCH collaborator:*** Peter Bugert.

***ESTHER collaborators:*** Volker Arndt, Heiko Müller, Christa Stegmaier.

***GENICA Network collaborators***: Wing-Yee Lo, Christina Justenhoven, Ute Hamann, Thomas Brüning, Beate Pesch, Yon-Dschun Ko, Sylvia Rabstein, Anne Lotz, Christina Baisch, Hans-Peter Fischer, Volker Harth.

## F. Supplementary Acknowledgements

The authors thank the many individuals who participated in this study and the numerous institutions and their staff who have supported recruitment.

ANECS thanks members of the Molecular Cancer Epidemiology and Cancer Genetic laboratories at QIMR Berfhofer Medical Research Institute for technical assistance, and the ANECS research team for assistance with the collection of risk factor information and blood samples. ANECS also gratefully acknowledges the cooperation of the following institutions: NSW: John Hunter Hospital, Liverpool Hospital, Mater Misericordiae Hospital (Sydney), Mater Misericordiae Hospital (Newcastle), Newcastle Private Hospital, North Shore Private Hospital, Royal Hospital for Women, Royal Prince Alfred Hospital, Royal North Shore Hospital, Royal Prince Alfred Hospital, St George Hospital; Westmead Hospital, Westmead Private Hospital; Qld: Brisbane Private Hospital, Greenslopes Hospital, Mater Misericordiae Hospitals, Royal Brisbane and Women's Hospital, Wesley Hospital, Queensland Cancer Registry; SA: Adelaide Pathology Partners, Burnside Hospital, Calvary Hospital, Flinders Medical Centre, Queen Elizabeth Hospital, Royal Adelaide Hospital, South Australian Cancer Registry; Tas: Launceston Hospital, North West Regional Hospitals, Royal Hobart Hospital; Vic: Freemasons Hospital, Melbourne Pathology Services, Mercy Hospital for Women, Royal Women's Hospital, Victorian Cancer Registry; WA: King Edward Memorial Hospital, St John of God Hospitals Subiaco & Murdoch, Western Australian Cancer Registry.

SEARCH thanks the SEARCH research team for recruitment, and also acknowledges the assistance of the Eastern Cancer Registration and Information Centre for subject recruitment.

BECS thanks Reiner Strick, Silke Landrith and Sonja Oeser for their logistic support during the study.

CAHRES (formerly known as SASBAC) thanks Li Yuqing from the Genome Institute of Singapore for contributions to this study, and also acknowledges previous input to SASBAC resource creation by Anna Christensson, Boel Bissmarck, Kirsimari Aaltonen, Karl von Smitten, Nina Puolakka, Christer Halldén, Lim Siew Lan and Irene Chen, Lena U. Rosenberg, Mattias Hammarström, and Eija Flygare.

HJECS thanks Wen Zheng, Hermann Hertel, and Tjoung-Won Park-Simon at Hannover Medical School for their contribution to sample recruitment.

LES gratefully acknowledges Helena Soenen, Gilian Peuteman and Dominiek Smeets for their technical assistance.

MECS thanks Tom Sellers, Catherine Phelan, Andrew Berchuck, and Kimberly Kalli, Amanda von Bismarck, Luisa Freyer and Lisa Rogmann.

MoMaTEC thanks Britt Edvardsen, Ingjerd Bergo and Mari Kyllsø Halle for technical assistance and Inger Marie Aksnes and Tor Audun Hervig at the Blood bank, Haukeland University Hospital for assistance with control recruitment.

NECS thanks staff at the University of Newcastle and the Hunter Medical Research Institute.

NSECG thank Ella Barclay and Lynn Martin for their contribution, and acknowledge the invaluable help of the National Cancer Research Network with the collection of study participants.

QIMR Berghofer thanks Margie Wright, Lisa Bowdler, Sara Smith, Megan Campbell and Scott Gordon for control sample collection and data processing, Kerenaftali Klein for statistical advice and Brendan Ryan for assistance with the figures.

RENDOCAS thanks Berith Wejderot, Sigrid Sahlen, Tao Liu, Margareta Ström, Maria Karlsson, and Birgitta Byström for their contribution to the study.

SECGS thanks research staff and participants of the Shanghai Endometrial Cancer Study and Shanghai Breast Cancer Study for their contributions in the study.

BSUCH thanks the Medical Faculty, Mannheim, the Diemtmar Hopp Foundation and the German Cancer Research Center.

MCCS was made possible by the contribution of many people, including the original investigators and the diligent team who recruited the participants and who continue working on follow up. We would like to express our gratitude to the many thousands of Melbourne residents who continue to participate in the study.

The UKBGS thank Breakthrough Breast Cancer and the Institute of Cancer Research for support and funding, and the Study participants, Study staff, and the doctors, nurses and other health care staff and data providers who have contributed to the Study. The ICR acknowledges NHS funding to the NIHR Biomedical Research Centre.

In addition, the iCOGS study would not have been possible without the contributions of: Andrew Berchuck (OCAC), Rosalind A. Eeles, Ali Amin Al Olama, Zsofia Kote-Jarai , Sara Benlloch (PRACTICAL), Georgia Chenevix-Trench, Antonis Antoniou, Lesley McGuffog, Fergus Couch and Ken Offit (CIMBA), Andrew Lee, and Ed Dicks, Craig Luccarini and the staff of the Centre for Cancer Genetic Epidemiology Laboratory (Cambridge), Javier Benitez, Anna Gonzalez-Neira and the staff of the CNIO genotyping unit, Jacques Simard and Daniel C. Tessier, Francois Bacot, Daniel Vincent, Sylvie LaBoissière and Frederic Robidoux and the staff of the McGill University and Génome Québec Innovation Centre, Stig E. Bojesen, Sune F. Nielsen, Borge G. Nordestgaard, and the staff of the Copenhagen DNA laboratory, Sharon A. Windebank, Christopher A. Hilker, Jeffrey Meyer and the staff of Mayo Clinic Genotyping Core Facility.

## H. Additional Acknowledgments of Funding to BCAC/OCAC control groups

The ESTHER study was funded by the Baden-Württemberg state Ministry of Science, Research and Arts (Stuttgart, Germany), the Federal Ministry of Education and Research (Berlin, Germany) and the Federal Ministry of Family Affairs, Senior Citizens, Women and Youth (Berlin, Germany).

The GENICA was funded by the Federal Ministry of Education and Research (BMBF) Germany grants 01KW9975/5, 01KW9976/8, 01KW9977/0 and 01KW0114, the Robert Bosch Foundation, Stuttgart, Deutsches Krebsforschungszentrum (DKFZ), Heidelberg, Institute for Prevention and Occupational Medicine of the German Social Accident Insurance, Institute of the Ruhr University Bochum (IPA), Germany, as well as the Department of Internal Medicine, Evangelische Kliniken Bonn gGmbH, Johanniter Krankenhaus, Bonn, Germany.

Financial support for the KARBAC study was provided through the regional agreement on medical training and clinical research (ALF) between Stockholm County Council and Karolinska Institutet, as well as the Swedish Cancer Society.

The MARIE study was supported by the Deutsche Krebshilfe e.V. [70-2892-BR I], the Hamburg Cancer Society and the German Cancer Research Center.

MAY was supported by R01-CA122443, P50-CA136393, the Fred C. and Katherine B. Andersen Foundation, and the Mayo Foundation.

MCBCS recognizes funding from the Breast Cancer Research Foundation (BCRF), the David F. and Margaret T. Grohne Family Foundation, and the Ting Tsung and Wei Fong Chao Foundation

MCCS recruitment was funded by VicHealth and Cancer Council Victoria, and its follow-up has been continuously supported by infrastructure provided by Cancer Council Victoria.

UKBGS was funded by Breakthrough Breast Cancer and the Institute of Cancer Research, which acknowledges NHS funding to the NIHR Biomedical Research Centre.
