# Supplementary material for: Fine-mapping of the HNF1B multicancer locus identifies candidate variants that mediate endometrial cancer risk
Source: Hum Mol Genet. 2014 Nov 6;24(5):1478–92. doi: 10.1093/hmg/ddu552 (PMC4321445; doi:10.1093/hmg/ddu552)
Supplement: Supplementary Data [file supp_ddu552_ddu552supp_table5.docx]

**Supplementary Table 5**. Summary of *in silico* transcription factor binding predictions using is-rSNP* for the five SNPs most strongly associated with endometrial cancer.

| **SNP** | **Variation**  **(Ref/Alt)^a^** | **Matrix^b^** | **Predicted TF^c^** | **Adjusted**  ***P*-value^d^** | **Evidence of TF implicated in endometrial cancer** |
| --- | --- | --- | --- | --- | --- |
| rs11263763 | G/A | M00157  M00034  M00480  M00188  M01331 | RORalpha2  p53  TOPORS  AP1  ISX | 0.00039  0.00051  0.00055  0.00056  0.00067 | Garg et al, Mod Pathol, 2010  Dube et al, Cancer Lett, 2009 |
| rs11651052 | A/G | M01408  M00465  M01124  M01469  M01723  M01650 | POU4F3  POU6F1  Oct-4  NKX6.1  SATB1  PNR | 0.00031  0.00046  0.00052  0.00058  0.00068  0.00088 | Wu *et al*, Cancer, 2011  Mokhtar *et al*, Cancer Invest, 2012 |
| rs8064454 | A/C | M00457  M00459  M00184  M01823  M01476  M00225  M00804  M00414 | STAT5A  STAT5B  MyoD  STAT1  POU2F3  STAT3  E2A  ZEB1 | 0.00013  0.00025  0.00045  0.00046  0.00071  0.00074  0.00091  0.00092 | Sharma *et al*, Endo Relat Cancer 2006  Spoelstra *et al*, Cancer Res, 2006 |
| rs10908278 | T/A | M00133  M01345  M0313  M01449  M01433  M01358  M0137  M00156  M00460  M01666  M01125 | Tst1  Six6  Six1  Cdx2  Six2  Six3  Cdx1  RORalpha1  STAT5A  STAT4  Oct-4 | 0.00011  0.00015  0.00017  0.00026  0.00042  0.00050  0.00063  0.00073  0.00077  0.00093  0.00096 | Wani et al, Hum Pathol, 2008  Wu *et al*, Cancer, 2011 |
| rs11651755 | C/T | M00225  M00224  M01299  M01001  M00420  M01112  M00034 | STAT3  STAT1  MECP2  DEAF1  HOXA9  RBPJ  p53 | 0.000018  0.000021  0.00041  0.00044  0.00080  0.00085  0.00099 | Chu et al, Cell Mol Life Sci, 2014  Garg et al, Mod Pathol, 2010 |

* Macintyre, G *et al*. Bioinformatics. 26(18):i524-30

^a^ Ref=reference allele, Alt=alternative allele; ^b^ Transcription factor (TF) matrix ID from the TRANSFAC database; ^c^ TF predicted to be disrupted; ^d^ Bonferroni corrected *P*-value of the observed difference between Ref and Alt allele *P*-values. Only results that show a significant (BH corrected P < 0.001) change in TF binding affinity between the alleles are included.

**References**

# Chu, Y *et al*. Chromatin composition alterations and the critical role of MeCP2 for epigenetic silencing of progesterone receptor-B gene in endometrial cancers. Cell Mol Life Sci. 71(17):3393-408. 2014

Dube, C *et al.* The nuclear receptors SF1 and LRH1 are expressed in endometrial cancer cells and regulate steroidogenic gene transcription by cooperating with AP-1 factors. Cancer Lett. 275(1):127-38. 2009

Garg, K *et al.* p53 overexpression in morphologically ambiguous endometrial carcinomas correlates with adverse clinical outcomes. Mod Pathol. 23(1):80-92. 2010

Macintyre, G *et al*. is-rSNP: a novel technique for in silico regulatory SNP detection. Bioinformatics. 26(18):i524-30. 2012

# Mokhtar, NM *et al*. Laser capture microdissection with genome-wide expression profiling displayed gene expression signatures in endometrioid endometrial cancer. 30(2):156-64 Cancer Invest, 2012

# Sharma, D *et al*. Leptin promotes the proliferative response and invasiveness in human endometrial cancer cells by activating multiple signal-transduction pathways. Endo Relat Cancer. 13(2):629-40. 2006

# Spoelstra, NS *et al*. The transcription factor ZEB1 is aberrantly expressed in aggressive uterine cancers. Cancer Res. 66(7):893-902. 2006

# Wani, Y *et al*. Aberrant Cdx2 expression in endometrial lesions with squamous differentiation: important role of Cdx2 in squamous morula formation. Hum Pathol. 39(7):1072-9. 2008

# Wu, Y *et al*, Up-regulation of microRNA-145 promotes differentiation by repressing OCT4 in human endometrial adenocarcinoma cells. Cancer. 117(17):3989-98. 2011
